# Loss of offspring *Peg3* reduces neonatal ultrasonic vocalizations and increases maternal anxiety in wild-type mothers 

**DOI:** 10.1093/hmg/ddx412

**Published:** 2017-11-24

**Authors:** G I McNamara, H D J Creeth, D J Harrison, K E Tansey, R M Andrews, A R Isles, R M John

**Affiliations:** 1 Biomedicine Division, School of Biosciences, Cardiff University, Cardiff CF10 3AX, UK; 2 MRC Centre for Neuropsychiatric Genetics and Genomics, School of Medicine, Cardiff University, Cardiff, UK; 3 Core Bioinformatics and Statistics Team, College of Biomedical & Life Sciences; 4 Systems Immunity University Research Institute, Cardiff University, Cardiff CF10 3XQ, UK

## Abstract

Depression and anxiety are the most common mental health conditions during pregnancy and can impair the normal development of mother-infant interactions. These adversities are associated with low birth weight and increased risk of behavioural disorders in children. We recently reported reduced expression of the imprinted gene *PATERNALLY EXPRESSED GENE 3* (*PEG3)* in placenta of human infants born to depressed mothers. Expression of *Peg3* in the brain has previously been linked maternal behaviour in rodents, at least in some studies, with mutant dams neglecting their pups. However, in our human study decreased expression was in the placenta derived from the fetus. Here, we examined maternal behaviour in response to reduced expression of *Peg3* in the feto-placental unit. Prenatally we found novelty reactivity was altered in wild-type females carrying litters with a null mutation in *Peg3*. This behavioural alteration was short-lived and there were no significant differences the transcriptomes of either the maternal hypothalamus or hippocampus at E16.5. In contrast, while maternal gross maternal care was intact postnatally, the exposed dams were significantly slower to retrieve their pups and displayed a marked increase in anxiety. We also observed a significant reduction in the isolation-induced ultrasonic vocalizations (USVs) emitted by mutant pups separated from their mothers. USVs are a form of communication known to elicit maternal care suggesting *Peg3* mutant pups drive the deficit in maternal behaviour. These data support the hypothesis that reduced placental *PEG3* in human pregnancies occurs as a consequence of prenatal depression but leaves scope for feto-placental *Peg3* dosage, during gestation, influencing aspects of maternal behaviour.

## Introduction

A recent UK study estimated that perinatal mental health problems cost the UK £8.1 billion each year with nearly three-quarters of this cost related to adverse impacts on the child (1). Depression and anxiety are the most common mental health conditions associated with pregnancy, estimated to affect as many as one in five women (2,3) with the highest risk in women with a history of mental illness and those exposed to adverse circumstances (4). Prenatal depression and anxiety are risk factors for post-natal depression (5) and can impair the normal development of post-natal maternal behaviour and mother-infant interactions (6). Prenatal depression is highly correlated with low birth weight (7–14), and both prenatal and post-natal depression are linked to neurodevelopmental problems in children (15–18). A number of other common conditions such as pre-pregnancy obesity, gestational diabetes and hypertension-spectrum pregnancy disorders co-occur with depression symptoms (19,20) and these are also associated with adverse outcomes for children (21–23). These co-occurrences add a high level of complexity to studying the causes and consequences of mental health conditions in human pregnancies.

Recently, we reported lower expression of the imprinted *PATERNALLY EXPRESSED GENE 3* (*PEG3*) in term placenta from women with either clinically diagnosed depression in pregnancy or questionnaire identified depression near term (24). *PEG3* is a paternally expressed imprinted gene whose allelic expression is established and maintained by DNA methylation spanning its promoter (25). *PEG3* encodes a zinc finger protein that represses the transcription of a number of genes involved in cellular metabolism (26,27). Studies in rodents have identified function for *Peg3* in regulating fetal and placental growth with loss-of-function resulting in low birth weight and lighter placenta (28–30). In adult mice, loss-of-function of *Peg3* has both metabolic (31) and behavioural consequences (28,32–34). As adults, *Peg3* mutant mice of both genders deposit excess fat despite consuming less food, potentially due to a lowered metabolic rate (31). Female *Peg3* mutant mice are slower to enter puberty (31) and, as adults, exhibit a deficit in classic tests of maternal behaviours, such as nest building and pup retrieval, alongside a defect in oxytocin-expressing neurons (28). Male *Peg3* mutant mice fail to respond normally to sexually receptive females (35). More recent observations suggest the maternal behaviour may be strain sensitive (30).

Behavioural studies have examined the consequences of loss-of-function of *Peg3* in the adult whereas we observed reduced expression in the placenta, which is derived from the fetus. No other study that has reported on maternal behaviour of wild-type (WT) dams carrying and nursing Peg3 KO offspring. Li *et al.* (1999) reported on the maternal behavioural phenotype of Peg3 KO dams mothering phenotypically WT pups (carrying inactive maternal allele) (28). Interbreeding of Peg3 heterozygotes mutant males and females resulted in a loss of litters but this also occurred in crosses between mutant females and WT males ie the genotype of the father and mutant phenotype of Peg3 offspring (50% would be KO) were not relevant for survival, at least in this study. Curley *et al.* (2004) reported on crosses between homozygous Peg3 KO males and WT females (32). Mutant litters had fewer pups that survived the first day of birth and mean pup weight was lower in Peg3 KO litters which could indicate suboptimal mothering, However, Peg3 KO pups gained less weight over the next 28 days than WT pups despite both groups being suckled by WT mothers, and this weight difference increased over time into the post-weaning period suggesting intrinsic growth restriction. *PEG3* is expressed and imprinted in the villous cytotrophoblast layer of the human placenta (36). The placenta transports nutrients and oxygen to the growing fetus, removes waste products and mitigates the mother’s immune response to her semi-allogeneic fetus as well as manufacturing large quantities of hormones that induce the adaptations required for a successful pregnancy (37). Placental dysfunction consequently can have a myriad of direct consequences including low birth weight potentially contributing to to neurodevelopmental abnormalities in children. Although loss-of-function of *Peg3* in mice results in low birth weight, this occurred in mixed litters of mutant and WT pups whereas term placental expression of *PEG3* in humans does not appear to correlate with birth weight in singleton pregnancies (24,38,39). In addition to supporting fetal growth, the placenta can provide a record of exposures during pregnancy, as evidenced by changes in the placental transcriptome and epigenome in response to a number of adversities (40). Such exposures may alter the function of the placenta contributing to placental dysfunction and adverse outcomes. Few studies have assessed the absolute expression levels of *PEG3* in relation to human conditions. However, DNA methylation can be used as a proxy for gene expression levels. Altered DNA methylation, primarily studied in infant cord blood samples, has been reported in association with infant temperament (greater externalizing and negative affectivity) (41) but not prenatal maternal stress (Perceived Stress Scale) (42), depressed maternal mood early in pregnancy (Centers for Epidemiologic Studies Depression Scale) or low birth weight (14). A number of factors have been linked to altered DNA methylation at *PEG3* including fetal alcohol syndrome (43), maternal antibiotic use (44), obesity (45), toxic metals (46), paternal obesity (47) and maternal folate intake (48), all of which suggest imprinting of *PEG3* is sensitive to environmental exposures.

We observed a 20–45% reduction in the expression of *PEG3* in term placenta from women with depressive symptoms in pregnancy in three independently recruited human cohorts (24). One of the greatest challenges of interpreting placental changes in gene expression in human pregnancies is establishing cause and effect relationships, and we were not able to establish whether reduced expression occurred as a cause or a consequence of the depression. Here, we sought to address this challenge by making use of a *Peg3* mutant mouse model. Transmission of a targeted *Peg3* allele via the paternal germline results in offspring with global loss-of-function of *Peg3* (28). We examined WT dams bearing litters of 100% *Peg3* mutant pups and found that the loss of offspring *Peg3* influenced maternal behaviour in the pre- and post-natal period.

## Results

**Figure 1.**
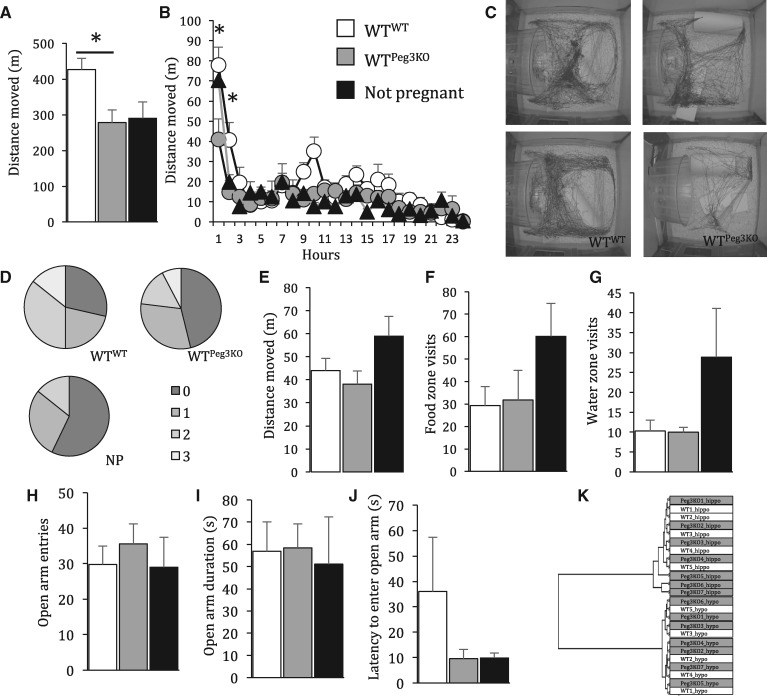
Carrying a litter null for *Peg3* (Peg3KO) has consequences for the wild-type (WT) dams’ novelty reactivity mid-gestation that normalises in late gestation. (**A**) WT^WT^ dams moved significantly more than WT^Peg3KO^ dams in the first 24 in a novel home cage that was apparent in the first 2 h (**B**). (**C**) Example activity tracing from the first 2 h in a novel home cage. (**D**) Nest quality score made by WT^WT^, WT^Peg3KO^ and not pregnant (NP) females. There was no effect of carrying a Peg3KO litter at E16.5 on distance moved (**E**), food (**F**) and water (**G**) zone visits during a nest building task. At E17.5 there was no difference between WT^WT^ and WT^Peg3KO^ females in number of (**H**), time spent on (**I**) and latency to enter (**J**) the anxiogenic open arms of an EZM. (**K**) hierarchical cluster of RNASeq data from the E16.5 hypothalamus and hippocampus indicates no difference between the transcriptome of WT^WT^ and WT^Peg3KO^ females. Data show mean ±SEM. *P < 0.05.

Fourteen days following observation of a vaginal plug, dams were individually housed in modified home cages to monitor behaviour longitudinally. In the first 24 h after separation from the male, WT dams carrying *Peg3*^+/^^−^ (inheritance of targeted paternal allele; Peg3KO) fetuses moved significantly less that WT dams carrying WT fetuses (*F*_2,__28_ = 4.739, *P* = 0.018, Dunnet post hoc WT^Peg3KO^ vs WT^WT^*P* = 0.011; Fig. 1A). This was not due to a difference in weight at 14.5 [WT^WT^ average 29.0 SEM 0.5 g, WT^Peg3KO^ average 29.1 SEM 0.6, *t*(23) = −0.104, *P* = 0.92]. This was particularly pronounced in the first hour in the novel arena, whereby WT^Peg3KO^ dams travelled significantly less distance than WT^WT^ (*F*_2,__28_ = 4.097, *P* = 0.028, Dunnett’s post hoc *P* = 0.023) dams and tended to travel less distance than not pregnant females (Dunnett’s post hoc *P* = 0.084; Fig. 1B and C).

To further assess pregnant dam behaviour nest building performance was assayed. The existing nest was removed and dams were provided with new nesting material. Increased nest building behaviour is a robust phenotype associated with pregnancy, the frequency of which increases towards parturition (49). In this assay, we found no gross differences in quality of nest built after 1 h between WT^WT^, WT^Peg3KO^ dams and non-pregnant females [χ(2) = 3.123, *P* = 0.21; Fig. 1D]. Pregnant dams tended to move less (*F*_2,__33_ = 2.339, *P* = 0.113) and make less visits to the food (*F*_2,__33_ = 1.553, *P* = 0.228) and water zone (*F*_2,__33_ = 2.863, *P* = 0.072) compared with non-pregnant females. However, this did not reach statistical significance (Fig. 1E–G). In a late gestation task of anxiety-related behaviour there was no difference between WT^WT^, WT^Peg3KO^ dams and non-pregnant females in any measures of the elevated zero maze (EZM), frequency of entries to (*F*_2,__32_ = 0.353, *P* = 0.706), duration of time spent on (*F*_2,__32_ = 0.056, *P* = 0.946) and latency to enter (*F*_2,__32_ = 0.991, *P* = 0.383) anxiogenic open arms (Fig. 1H–J). Reflecting this similarity in behaviour later in gestation, at E16.5, there was no significant difference between the transcriptome of WT^WT^ and WT^Peg3KO^ dams in either the hippocampus or hypothalamus, as demonstrated by the overlap in hierarchical clustering within brain regions (Fig. 1K). This suggests a subtle difference in behaviour, in response to a novel arena, of WT dams carrying Peg3KO fetuses in mid-gestation.

**Figure 2.**
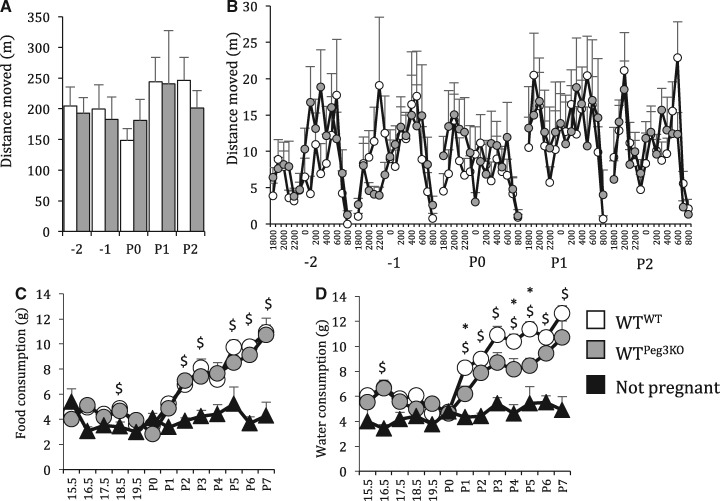
Water, but not food, consumption is reduced in lactating WT^Peg3KO^ females. (**A**) There was no difference in distance moved in five days around parturition and (**B**) normal diurnal activity was intact in WT^Peg3KO^ as indicated by hourly distance moved between 1800 and 0800 h. (**C**) Food consumption increased in lactating dams compared with mated females that did not become pregnant (NP). (**D**) Water consumption increased during lactation but this was less pronounced in WT^Peg3KO^ dams. Data show mean ±SEM. * WT^WT^ significantly different from WT^Peg3KO^ $WT^WT^ significantly different from not pregnant, one-way ANOVA Dunnet post hoc test.

Unlike locomotor activity during habituation to the novel home cage, dam activity levels in the five days around parturition were not different between WT^WT^ and WT^Peg3K^^O^ (see Supplementary Material, Table S2 for statistical details; Fig. 2A and B). Previously it has been reported that Peg3KO fetuses were lighter than their WT littermate controls at E17.5 (28). In the single genotype litters in this study, Peg3KO fetuses were not lighter that WT fetuses from the concurrently studied fully WT controls at E16.5 [*t*(88) = −0.792, *P* = 0.431; Supplementary Material, Fig. S1A]. Peg3KO placenta were significantly lighter than WT at E16.5 [*t*(87) = 9.788, *P* < 0.001; Supplementary Material, Fig. S1B]. Peg3KO pups are already known to be significantly lighter than their WT littermates (28,32), and we found that single genotype litters were lighter at P7 [*t*(131) =  8.892, *P* < 0.001; Supplementary Material, Fig. S1C]. Gestation length was also normal in WT^Peg3KO^ dams (*F*_1,__24_ 0.025, *P* = 0.876; Supplementary Material, Fig. S1D). Food consumption increased significantly from P2 onwards in lactating dams in comparison to non-pregnant females (see Supplementary Material, Table S3 for statistical details; Fig. 2C). A similar pattern was observed for water consumption during lactation (Fig. 2D). There was a significant effect of lactation on water consumption from P1. WT dams nursing Peg3KO pups consumed significantly less water than WT^WT^ dams on P1, 4 and 5. This was not a factor of different number of pups being nursed as litter size was equivalent between the groups [*t*(26) = −0.110, *P* = 0.913; Supplementary Material, Fig. S1E].

**Figure 3.**
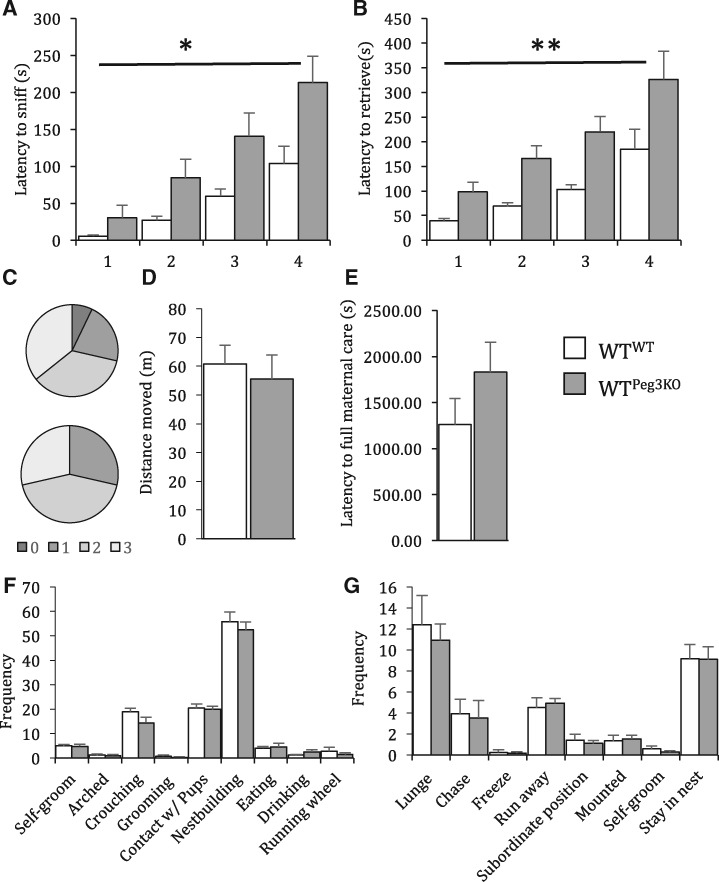
Exposure to Peg3KO pups increases WT dam latency to sniff and retrieve. Latency to sniff (**A**) and retrieve (**B**) pups. (**C**) Nest quality score on P3. There was no difference in distance moved (**D**), latency to full maternal care (**E**) time spent on nest on P1 (**F**) or maternal/nest building behaviour (**G**) on P3. There was no difference in aggression displayed towards an intruder male on P7 between WT^WT^ and WT^Peg3KO^ dams (**H**). Data show mean ±SEM. *P < 0.05 **P < 0.01.

Neonatal pups cannot thermoregulate and, as such, require maternal crouching to maintain body temperature. When pups are displaced from the nest, dams will rapidly return pups (50). This behaviour has been shown to be impaired in dams lacking *Peg3* (33). In this study, the time taken by WT dams exposed to WT or Peg3KO pups to sniff and retrieve four of their own pups was recorded for each dam on P2. WT dams nursing Peg3KO pups were significantly slower to sniff (*F*_1,__25_ = 6.41, *P* = 0.018) and retrieve (*F*_1,__25_ = 11.11, *P* = 0.003) pups (Fig. 3A and B). This was not associated with any difference in nest quality on P3 [χ(1) = 0.021, *P* = 0.884; Fig. 3C], nor in activity during a nest building task (*F*_1,__27_ = 0.26, *P* = 0.62; Fig. 3D). While building a nest, there is a conflict between the time spent by the dams spend building a nest and the time spent crouching over the litter to maintain pup body temperature. There was no difference between WT^WT^ and WT^Peg3KO^ dams in latency to display full maternal care, defined as crouching over all pups and building a nest (*F*_1,__27_ = 1.87, *P* = 0.18; Fig. 3E). Dam behaviour during this task was scored for nest building-related and pup directed behaviours. None of these behaviours were different between dams nursing WT or Peg3KO pups, indicating that the increased latency to sniff and retrieve pups was not associated with gross deficits in maternal care (Fig. 3F, All *P* > 0.05; See Supplementary Material, Table S2 for statistical details). Furthermore, there was no difference in duration of time spent on the nest in on P1 [*t*(23) = 0.6542, *P* = 0.52] This was emphasised by no difference in aggressive and defensive behaviours towards an intruder male on P7 (Fig. 3G, all *P* > 0.05; See Supplementary Material, Table S3 for statistical details).

**Figure 4.**
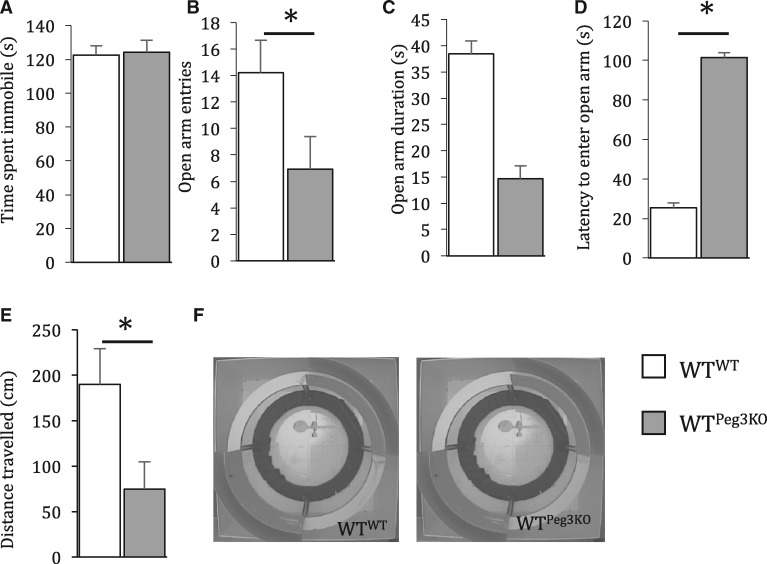
Anxious- but not depressive-like behaviours are increased in dams nursing Peg3KO pups. (**A**) Time spent immobile in a tail suspension task. (B-E) In an elevated zero maze WT^Peg3KO^ females made fewer entries (**B**), spent less time on (**C**), were slower to enter (**D**) and (**E**) travelled a shorter distance in the open arms of the EZM. (F) Example activity traces made by WT^WT^ and WT^Peg3KO^ females. Data show mean ±SEM. *P < 0.05.

Given the association between low placental *PEG3* expression and maternal prenatal depression in humans, we assessed anxiety behaviour in EZM test and time spent immobile in a tail suspension test as a proxy of ‘mood’. While no difference was observed in time spent immobile in the tail suspension test between WT^WT^ and WT^Peg3KO^ dams (*F*_1,__20_ = 0.032, *P* = 0.86; Fig. 4A), WT^Peg3KO^ dams displayed significantly greater anxious behaviour than WT^WT^ dams. WT^Peg3KO^ dams made significantly less entries to (*F*_1,__27_ = 4.51, *P* = 0.044), were significantly slower to enter (*F*_1,__27_ = 4.47, *P* = 0.045) and travelled a shorter distance (*F*_1,__27_ = 5.13, *P* = 0.032) in the open arms (Fig. 4B–F). This suggests that a consequence of carrying and nursing Peg3KO pups is increased anxiety in a WT dam or, alternatively, a failure to show the normal lactation-associated decrease in anxiety.

**Figure 5.**
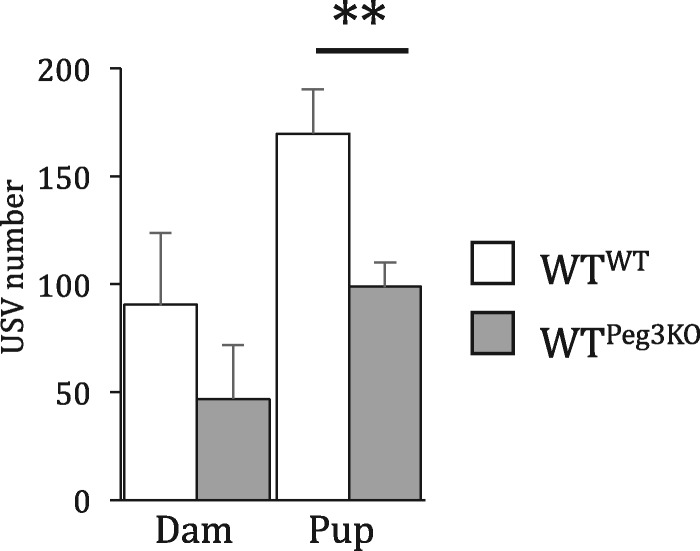
Mutation in *Peg3* reduced the number of isolation-induced USVs. Isolation-induced USV made by dams and four pups. Data show mean ±SEM. **P < 0.01.

During periods of maternal separation from pup, both the lactating female and the neonatal pups will vocalize in the ultrasonic range. This is thought to communicate location and arousal state information between dam and pup (51). Calls made by dams and pups were recorded separately. Peg3KO pups, in the absence of the dam, made significantly less USVs compared with WT pups [*t*(24) = 3.035, *P* = 0.006; Fig. 5]. Similar to intact maternal behaviours in the nest building and resident intruder tasks, WT^Peg3KO^ dams did not differ significantly from their WT^WT^ counterparts in USV number [*t*(24) = 0.895, *P* = 0.38]. These data suggest that, while gross maternal care is intact in WT dams nursing PegKO pups, a consequence of this exposure is heightened anxiety in the dams.

## Discussion

We recently reported an association between reduced placental *PEG3* expression and prenatal depression in human mothers (24). This raised the possibility that reduced placental *PEG3* might contribute to the programming of prenatal depression. To explore this, we used an experimental system where the behaviour of female mice exposed to a lower dose of *Peg3 in utero*. Apart from a subtle effect on exploratory behaviour by pregnant dams in a novel arena, our data did not support a causal role for reduced *Peg3* in the programming prenatal brain and behaviour. However, post-natally mothers that carried exclusively Peg3KO pups displayed reduced retrieval of pups and increased general anxiety. In addition, we identified a very early defect in vocalization in Peg3KO pups. Taken together, these data suggest a functional role for reduced *Peg3* dosage in the offspring in influencing maternal behaviour, with important relevance for maternal post-natal anxiety.

In mice, *Peg3* regulates fetal and placental growth (28,29). Loss-of-function has been linked to altered sexual behaviour in males (35) and deficient maternal care behaviour in females (28), at least in some studies (30). *Peg3* regulates a network of 22 genes concerned with neural development (52). Together, these data suggest *Peg3* as a promising candidate for human behavioural disorders. However, the association we reported between reduced *PEG3* expression and prenatal depression in human mothers was in the fetally-derived placenta (24). Reduced placental *PEG3* might contribute to the programming of prenatal depression in the mother, potentially by alternating the endocrine function of the placenta. Alternatively, lower *PEG3* could occur in response to the maternal depression or associated lifestyle factors. To explore these relationships, in the present study, we examined the behaviour of female mice exposed to a lower dose of *Peg3 in utero* in comparison to control females. In this model, *Peg3* is expressed in the dams at a normal level. We found a subtle difference in reactivity to novelty in dams carrying embryos null for *Peg3*, specifically on E14.5 WT^WT^ dams travelled significantly further than WT^Peg3KO^ dams during the first 24 h in a novel home cage. This can be attributed to novelty reactivity, rather than hypoactivity *per se*, as this difference manifests in the first hour and normalised over time. Furthermore, in the five days around parturition there was no difference in activity between WT^WT^ and WT^Peg3KO^ dams, excluding general hypoactivity as a confounding factor. Despite these changes, we found no evidence of prenatal anxiety in the EZM, and normal diurnal activity patterns and food consumption. These behaviours are deregulated frequently in depression in humans but were intact in WT^Peg3KO^ dams. We also examined gene expression in the hypothalamus (onset, maintenance and regulation of maternal behaviour) and the hippocampus (memory, learning and responses to fear and stress) (53) of dams. There were no significant differences in gene expression between the two cohorts of females at E16.5. Taken together, these data suggest a very subtle change in behaviour induced by carrying PegKO pups that are short-lived, suggesting that offspring *Peg3* has minimal impact on maternal behaviour pre-natally.

In contrast, we observed a robust phenotypic difference in the behaviour of WT^Peg3KO^ dams in the post-natal period. WT dams that carried and cared for litters of exclusively Peg3KO pups were slower to sniff and retrieve their pups (Fig. 3B and C), and displayed significantly greater anxiety in the EZM test (Fig. 4B and D). This was not associated with gross changes in maternal care as WT^Peg3KO^ dams constructed a nest and exhibited normal crouching behaviour. Presence of generally normal maternal care was supported by no difference in the display of maternal aggression towards an intruder male. However, this task was performed on P7 and, while this time point has been used in other studies on maternal behaviour (33), it is possible that differences in aggression may be apparent at an earlier timepoint when maternal behavioural phenotypes are most intense in rodents (54,55). As expected food consumption in the postpartum period was increased in the two pregnant groups relative to non-pregnant females, but was not significantly different between WT^WT^ and WT^PegKO^ females. WT^Peg3KO^ dams showed altered drinking behaviour. WT^Peg3KO^ females consumed significantly less water that their WT^WT^ counterparts, strictly in the postpartum period. This indicates that, while water consumption was reduced in WT^PegKO^ females, caloric intake was equivalent between groups.

From this study, it is not possible to completely disentangle whether the altered post-natal behaviour of WT^Peg3KO^ dams is due to abnormal placental function and aberrant maternal programming, or an altered post-natal environment due to the presence of Peg3KO pups. Our finding of reduced USVs by Peg3KO pups strongly supports the latter mechanism. Pups normally begin vocalizing, including ultrasonic whistles and clicks, shortly after birth with the rate of vocalizations peaking 7–8 days after birth. When separated from their mothers, USVs increase in rate and intensity, known as ‘whistles of loneliness’ (56). USVs are known to induce maternal behaviours such as nest building, pup retrieval and nursing (45,57–60). USVs are believed to initiate pup retrieval and once a pup is located, its retrieval is independent of USV (51). We asked whether the maternal behaviours in the WT mothers might be influenced USVs. We observed a 42% decrease in the USV number from the four pups that were used in the pup retrieval task during the period of separations. This corresponded with a 49% decrease in maternal vocalization, though this did not reach statistical significance. This suggests that the delay in pup retrieval may, in part, be due to reduced communication from the pups, a phenotype not previously reported for this mutation.

In mixed genotype litters, lethality associated with a *Peg3* mutation is higher than that observed in single genotype litters (29). This is suggested to be as a consequence of increased competition with WT pups. A reduced demand for maternal care via reduced USV by Peg3KO pups may also influence this outcome. Intriguingly, reduced pup USVs may also be linked to the marked change in anxiety behaviour seen in WT^Peg3KO^ dams. A lactation-associated reduction in anxiety has been observed both in rodents and humans, thought to facilitate offspring care (53,61). As we did not include mated but non-lactating controls in the anxiety assessment, we are unable to say that the increased anxiety in the WT^Peg3KO^ dams relative to the WT^WT^ dams is a failure to show the normal lactation-associated decrease in anxiety. However, the anxiolytic effect of lactation is known to be dependent on the activity of prolactin in the subventricular zone (61,62). Pup USV rapidly induces prolactin in lactating dams (63). Therefore, it is possible that the reduced USV production associated with a null mutation in offspring *Peg3* increases maternal anxiety in the post-natal period as well as reducing pup retrieval.

It is important to note that all these studies were performed on a 129 strain background. Differences in the maternal behavioural phenotype between dams mutant for *Peg3* have been reported which may be due to differences in strain background (28,30,32). However, in our study, the dams were genetically WT exposed to either genetically WT pups or *Peg3* mutant pups. We ensured that all our WT dams were from our in house 129 colonies, i.e. genetically and environmentally matched, and that the Peg3KO line was also maintained on this same 129 background. All our dams’ phenotypes can confidently be attributed specifically to the genetic manipulation in the pups. It may be that studying this genetic modification on a different strain background would reveal more information related specifically to depression but, nonetheless, we have shown for the first time that the pups’ *Peg3* expression status is important for maternal care.

Taken together, our data do not support our original idea, stemming from our previous study (24), that reduced placental expression of *PEG3* contributes to prenatal depression in human pregnancies. It seems more probable, given the number of factors thought to influence *PEG3* expression, that reduced placental *PEG3* occurs in response to depression, with potentially adverse consequences for post-natal maternal anxiety and infant neurodevelopment. However, these findings must be interpreted with caution. Firstly, while mice are useful in modelling genetic alterations reported in various human conditions, the mouse and human placenta differ considerably as does the hormonal repetoire of pregnancy (64). Secondly, in our human study, placental *PEG3* expression was reduced by a maximal 40% (24) and we do not know the *PEG3* expression status in the infant whereas in the animal model, *Peg3* expression is globally absent. Thirdly, while some aspects of human depression can be inferred from animal models including measures of helplessness, anhedonia, behavioural despair, alterations in sleep and appetite patterns (65), we were unable to use the full repertoire of behavioural assays because we were working with either pregnant or lactating females. Altered sleep patterns are a common feature of depression and we analysed circadian rhythmicity of activity over 5 days from two days before birth to P2 and did not find any difference in diurnal activity. We also analysed helplessness using the tail suspension task and did not find any evidence for a difference. However, for welfare reasons, we were unable to apply Lick-cluster analysis to measure anhedonia as used in adult non-pregnant mice (66) which may have provided additional weight to our findings. Finally, our human cohort identified lower *PEG3* expression only in male placenta. Human pregnancies are generally singleton but mice have litters with both male and female pups. This may preclude the identification of sexually dimorphic effects of the placenta on maternal behaviour.

In conclusion, carrying and caring for Peg3KO pups leads to a number of changes in post-natal maternal behaviour. Some of these may be due to abnormal maternal programming by *Peg3* expression in the fetally-derived placenta, although only subtle prenatal behavioural changes were found in WT^Peg3KO^ dams and no differences were seen in hypothalamic and hippocampal gene expression. A factor that may contribute to the changes in post-natal maternal behaviour is the reduced USVs produced by Peg3KO pups which may elicit less prominent retrieval behaviour from the mother. This is contrast to imprinted *Ube3a*, where pups lacking this maternally expressed gene have increased USVs (67), paralleling findings in individuals with Angelman syndrome where *UBE3A* is the key candidate (68). Reduced isolation-induced USVs have been reported in a number of genetic mouse models of neurodevelopmental disorders such as autism spectrum disorder (ASD), most recently reported for the *Foxp1* model (69). Prenatal depression has been linked to ASD, although several studies suggest this is related to prenatal antidepressant use (70). Given the association between maternal prenatal depression and reduced placental *PEG3* expression in humans, these findings have intriguing implications for low maternal mood, exacerbation of maternal anxiety and neurodevelopmental outcomes in offspring.

## Materials and Methods

### Animals and husbandry

All animal studies and breeding were approved by the University of Cardiff ethical committee and performed under a UK Home Office project license (RMJ). Mice were housed on a 12-h light–dark cycle with lights coming on at 07.00 h with a temperature range of 21 °C ± 2 with free access to tap water and standard chow. The *Peg3-*targeted allele (28), provided by Professor Azim Surani, was bred to homozygosity on the 129S2/SvHsd background, and all studies were performed on this background. Genotyping was performed using primers CGTTGGCTACCCGTGATATT and TATGCACACAGCCTCTGCTC or by *β-galactosidase* staining as previously described (71).

### Generation of experimental females

Litters were generated by natural mating *Peg3*^−^^/^^−^ males or wild males (WT) with WT females. Embryonic stage was calculated by vaginal plug check and the day of birth was considered as post-natal day (P)0. On embryonic day (E)14.5 females were removed from home cage, weighted and placed inside a Phenotyper cage (Noldus, Netherlands). Cages were equipped with cameras in the roof for video recording. Activity was tracked using Ethovision XT software (Noldus, Netherlands). A separate cohort of females were sacrificed at E16.5 and hippocampi and hypothalami were isolated, snap frozen and stored at −80 °C for later RNASeq analysis. Dams with litter sizes between 4 and 10 live pups were used for analysis. Pregnant group sizes WT^WT^*n* = 14 and WT^Peg3KO^*n* = 14. A subset of females mated but did not become pregnant. These were included as a ‘not pregnant’ reference group (*N* = 7).

### RNAseq

Total RNA was prepared from frozen mouse brain tissue samples using GenElute™ Mammalian Total RNA Miniprep Kit (Sigma, UK). RNA yield and quality [RNA integrity number (RIN)] were quantified using a Qubit 3.0 fluorometer. Input RNA yield ranged from 34 to 438 ng/μl. RIN values for all used were 8.5 or greater. Libraries were prepared with oligo dT priming using TruSeq Stranded mRNA Library Prep Kit (Illumina) and sequenced on an Illumina NextSeq500 using 75 base reads. Sequencing was performed in duplicate to normalise read depth to approximately 40 million reads per sample. Reads were aligned to the mouse genome (Mus_musculus.GRCm.38.88) with corresponding gene model annotation (Mus_musculus.GRCm38.88.gtf) by STAR (72). Overall read alignment rates were above 95% for all libraries. Differential gene expression was determined using DESeq2 1.0.19 (73).

### Behavioural assays

#### Nest building 

On E16.5 and P3 dams (and litter when present) were removed from the Phenotyper cage. Nest and bedding were discarded and replaced with three nestlets (Ancare, US) and one cardboard tube (International Product Supplies, UK). The litter was then returned to Phenotyper cage and video recording was initiated. Dams were reintroduced to the Phenotyper cage and allowed to build a nest. After 1 h, the quality of the nest was scored visually. This was a quantitative score determined by number of nestlets shredded to form a nest. A score from 0 to 3 was assigned each animal. Distance moved during this trial and visits to virtual ‘zones’ was calculated automatically using Ethovision XT software. At P3, maternal care and nest building behaviours were scored manually off line by a rater blind to genotype of the pups. Behaviours scored were arched nursing, licking and grooming of pups, contact with pups, nest building, self-grooming, eating and drinking. Latency to display maternal care was recorded; specifically this was determined to be latency to crouch over all pups and build and nest.

#### Elevated zero maze

On E17.5 and P4, dams were allowed to explore an EZM freely for 300 s. The EZM consisted of two open and two closed arms, in a circle 45 cm from the ground. Platform width was 5 cm and wall height in closed arms was 19 cm. Testing was carried out under low light conditions (230 lux on open sections) and all animals were placed in a closed arm at the beginning of a trial. Time spent in open arms, indicative of less anxious behaviour, was recorded automatically using Ethovision XT software.

#### Pup retrieval and ultrasonic vocalization recording

On P2 a dam’s litter was removed from the Phenotyper cage and placed in a standard home cage. Ultrasonic vocalizations (USVs) made by the dam for was recorded 180 s using Avisoft-UltraSoundGate 116Hb (Avisoft Bioacoustics e.K., Germany). The dam was then removed from the Phenotyper cage and placed in a standard home cage separate to pups. Where possible, two male and two female pups, determined visually by anogenital distance, were placed in Phenotyper cage opposite the nest and spaced apart, alternating male and female. USVs made by these four pups were simultaneously recorded for 180 s as above. Following this video recording was initiated and dam was reintroduced to the Phenotyper cage, indicating time zero. Time taken to sniff and retrieve each pup was recorded by experimenter and confirmed off line from video recordings. When all pups had been retrieved by the dam, remaining pups were returned to the Phenotyper.

#### Tail suspension

On P5 dams were removed from the Phenotyper cage for tail suspension testing. Dams had tape wrapped around their tail 1 cm from the tip of the tail, with 1 cm overlap of tape. The dam was suspended from a beam in front of a video recorder approximately 10 cm from nose to the surface. Trials lasted 300 s. Time spent immobile was scored manually offline.

#### Maternal aggression

Maternal aggression testing was carried out as described (33). Briefly, on P7 pups were removed from the Phenotyper cage, weighed and placed in a standard home cage. Video recording was started and a sexually experienced, vasectomised, male was introduced to Phenotyper cage containing the lactating dam. Behaviour of the dam was recorded manually offline by a scorer blind to the genotype of the litter. Behaviours scored were: Lunge at male, attack male, chase male, assume subordinate position (rear on hind legs, exposing underside), be mounted by male, self-grooming and staying on the nest.

### Statistical analysis

Excluding RNASeq analysis detailed above, all statistical analysis was carried out using SPSS 20. For analysis of prenatal behaviour between WT^WT^, WT^Peg3KO^ and mated but not pregnant females a series of one-way ANOVAs was carried out with Dunnett’s post hoc tests were appropriate (control group WT^WT^). For nest score analysis, a Kruskal-Wallis H test was performed on score categories. For analysis of food and drink consumption across pregnancy and the postpartum period a repeated measures ANOVA with DAY as the within subject variable, and LITTER SIZE as the covariable were performed. Post-natal behaviour between WT^WT^and WT^Peg3KO^ dams was measured by ANOVA with LITTER SIZE as the covariable, excepting pup retrieval that was analysed using repeated measures ANOVA with PUP as the within subject variable, and LITTER SIZE as the covariable.

## Supplementary Material

Supplementary Material is available at *HMG* online.

## Supplementary Material

Supplementary Tables
